# One-Stop Management of 230 Consecutive Acute Stroke Patients: Report of Procedural Times and Clinical Outcome

**DOI:** 10.3390/jcm8122185

**Published:** 2019-12-11

**Authors:** Marios-Nikos Psychogios, Ilko L. Maier, Ioannis Tsogkas, Amélie Carolina Hesse, Alex Brehm, Daniel Behme, Marlena Schnieder, Katharina Schregel, Ismini Papageorgiou, David S. Liebeskind, Mayank Goyal, Mathias Bähr, Michael Knauth, Jan Liman

**Affiliations:** 1 Department of Neuroradiology, University Medical Center Goettingen, 37075 Goettingen, Germany; ioannis.tsogkas@usb.ch (I.T.); amelie.hesse@med.uni-goettingen.de (A.C.H.); alex.brehm@usb.ch (A.B.); daniel.behme@med.uni-goettingen.de (D.B.); katharina.schregel@med.uni-goettingen.de (K.S.); michael.knauth@med.uni-goettingen.de (M.K.); 2 Department of Neuroradiology, Clinic for Radiology & Nuclear Medicine, University Hospital Basel, 4031 Basel, Switzerland; 3 Department of Neurology, University Medical Center Goettingen, 37075 Goettingen, Germany; ilko.maier@med.uni-goettingen.de (I.L.M.); marlena.schnieder@med.uni-goettingen.de (M.S.); mbaehr@gwdg.de (M.B.); jliman@gwdg.de (J.L.); 4 Department of Neuroradiology, Südharz Klinikum, 99734 Nordhausen, Germany; ismini.e.papageorgiou@gmail.com; 5 Neurovascular Imaging Research Core and Stroke Center, Department of Neurology, University of California Los Angeles, Los Angeles, CA 90095, USA; davidliebeskind@yahoo.com; 6 Calgary Stroke Program, Department of Clinical Neurosciences, University of Calgary, Calgary, AB 2500, Canada; mgoyal@ucalgary.ca

**Keywords:** stroke, hemorrhage, thrombectomy, cone-beam computed tomography, cerebral angiography

## Abstract

Background and purpose: Rapid thrombectomy for acute ischemic stroke caused by large vessel occlusion leads to improved outcome. Optimizing intrahospital management might diminish treatment delays. To examine if one-stop management reduces intrahospital treatment delays and improves functional outcome of acute stroke patients with large vessel occlusion. Methods: We performed a single center, observational study from June 2016 to November 2018. Imaging was acquired with the latest generation angiography suite at a comprehensive stroke center. Two-hundred-thirty consecutive adults with suspected acute stroke presenting within 6 h after symptom onset with a moderate to severe National Institutes of Health Stroke Scale (≥10 in 2016; ≥7 since January 2017) were directly transported to the angiography suite by bypassing multidetector CT. Noncontrast flat-detector CT and biphasic flat-detector CT angiography were acquired with an angiography system. In case of a large vessel occlusion patients remained in the angiography suite, received intravenous rtPA therapy and underwent thrombectomy. As primary endpoints, door-to-reperfusion times and functional outcome at 90 days were recorded and compared in a case-control analysis with matched prior patients receiving standard management. Results: A total of 230 patients (123 women, median age of 78 years (Interquartile Range (IQR) 69–84)) were included. Median symptom-to-door time was 130 min (IQR 70–195). Large vessel occlusion was diagnosed in 166/230 (72%) patients; 64/230 (28%) had conditions not suitable for thrombectomy. Median door-to-reperfusion time for M1 occlusions was 64 min (IQR 56–87). Compared to 43 case-matched patients triaged with multidetector CT, median door-to-reperfusion time was reduced from 102 (IQR 85–117) to 68 min (IQR 53–89; *p* < 0.001). Rate of good functional outcome was significantly better in the one-stop management group (*p* = 0.029). Safety parameters (mortality, sICH, any hemorrhage) did not differ significantly between groups. Conclusions: One-stop management for stroke triage reduces intrahospital time delays in our specific hospital setting.

## 1. Introduction

Swift and complete reperfusion of the occluded vessel territory is the key of every revascularization therapy in stroke patients with large vessel occlusion (LVO) [1,2]. Thrombectomy became the new standard of LVO-therapy after publication of multiple trials showing higher reperfusion rates and improved functional outcomes in patients receiving the combination of thrombectomy and medical therapy as opposed to medical therapy alone [3,4,5,6,7]. However, door-to-groin times have been consistently longer than one hour, even in trials focusing on rapid treatment of stroke patients [2,6]. The primary limitation leading to time-delays in the treatment of LVO-patients is the lack of a fast, reliable and affordable prehospital screening tool in stroke treatment akin to e.g., the electrocardiogram in patients with an acute coronary syndrome. While STEMI-patients with a positive electrocardiogram are directly transported to the angiography-suite, stroke patients are usually first triaged with a noninvasive imaging method in one room, or even hospital, and then transported to a different room, or even different hospital, for thrombectomy.

In order to minimize intrahospital times at the treating hospital, we recently proposed a method of noninvasive triage with a flat-detector computed tomography (FDCT) capable angiography-suite, IV lysis and thrombectomy in the same room (one-stop management) with the potential of significant reduction of door-to-groin and door-to-reperfusion times [8]. We demonstrated in prior work, that a rather simple, fast and commercially available non-enhanced FDCT protocol can be used to detect intracranial hemorrhage (ICH) with a very high sensitivity, which is comparable to multi-detector CT (MDCT) [9]. Furthermore, biphasic FDCT angiography enabled us to reliable detect LVOs and grade collaterals [10]. These advancements made the aforementioned paradigm feasible for the triage of mothership, who are eligible for IV lysis, as well as transfer patients.

In this study, we report the first 230 consecutive stroke patients diagnosed and treated with a one-stop management and analyze the 90 days functional outcomes, compared to patients managed with an optimized stroke workflow previously published [11].

## 2. Materials and Methods

### 2.1. Patient Selection

This observational study includes all 230 consecutive adult patients treated with one-stop management in our hospital from June 2016 to November 2018. All patients presented with clinical signs of an ischemic stroke within 6 h after symptom onset and a National Institutes of Health Stroke Scale (NIHSS) (≥10 in 2016; ≥7 since January 2017) were included in our study. Unknown symptom onset, prolonged time from symptom onset, a low NIHSS (<10 in 2016; <7 since January 2017) or occupation of the FDCT-capable angiography-suite during admission were exclusion criteria. Data were prospectively collected and documented in an Institutional Review Board-approved database. A neurological assessment was performed (i) at hospital admission, (ii) hospital discharge and (iii) 90 days after stroke by a certified stroke neurologist. The imaging data were documented by the treating physician and re-evaluated by a core-team, consisting of an experienced neurointerventionalist (>10 years of experience) and a neuroradiology resident. A patient’s consent for treatment was obtained according to the institutional guidelines. The local ethics committee waived the need for a formal application or a separate consent concerning the inclusion in our observational database.

### 2.2. Image Acquisition and Processing

Images were acquired using an Artis Q angiography system (Siemens Healthcare GmbH, Forchheim, Germany) as described before [8,12]. First, an FDCT was acquired to exclude intracranial hemorrhage. A commercially available 20 s rotational acquisition was used (20 s DCT Head, 109 kV, 1.8 μGy/frame, 200° angle, 0.4°/frame angulation step; effective dose ~2.5 mSv; Siemens Healthineers AG, Erlangen, Germany) and raw data were instantly and automatically reconstructed in 5 mm multiplanar reconstructions on a commercially available workstation (syngo × Workplace; Siemens Healthineers AG, Erlangen, Germany). Next, a commercially available biphasic FDCT-angiography (biFDCTA) was acquired for detection of arterial occlusion and evaluation of intracranial collaterals (2 × 10 s DSA, 70kV, 1.2 μGy/frame, 200° angle, 0.8°/frame angulation step; effective dose ~ 2.5 mSv; Siemens Healthineers AG, Erlangen, Germany) after intravenous injection of 60 mL contrast media (Imeron 400; Bracco Imaging Inc, Konstanz, Germany) at a flow rate of 5 mL/s followed by 60 mL saline chaser at 5 mL/s. Both FDCTA datasets were instantly and automatically reconstructed on the aforementioned workstation and 24 mm transversal maximal intensity projections of the first and second phase were simultaneously viewable on the workstation. Timing for the start of the first (arterial) phase acquisition was determined using a bolus-tracking acquisition. The second (venous) phase was acquired automatically with a delay of 5 s from the end of the first rotation. The acquisition, reconstruction and evaluation of all datasets do not require more than 2 min.

### 2.3. Management After Imaging

Patients with no hemorrhage and with an LVO were treated, if eligible, with intravenous recombinant tissue plasminogen activator (IV rtPA) and with thrombectomy. As per institutional guidelines, a low Alberta Stroke Program Early CT Scale (ASPECTS) or low collateral score was not an exclusion criterion for thrombectomy in the first 6 h after symptom onset. Patients with no hemorrhage and with a small vessel occlusion (SVO) were treated with IV rtPA only, if eligible. Patients with no hemorrhage and with no arterial occlusion were started on IV rtPA, if eligible, and received an additional stroke MRI to decide on further treatment. Patients with an intracranial hemorrhage and no occlusion were treated as per institutional standards. Lastly, patients with an intracranial hemorrhage (ICH) and LVO were treated with thrombectomy after an individualized case discussion between the neurologist, interventional neuroradiologist and patient or his/her next of kin.

### 2.4. Statistical Analysis

Characteristics and time-metrics of the one-stop database are reported by descriptive statistics. Time-intervals are documented with median, interquartile range (IQR) and 90th percentile, as recently proposed [13]. A case-matched analysis is performed between the one-stop database and the standard workflow (multidetector CT (MDCT)-triaged patients) database with the following criteria: patient’s age, admission NIHSS, ASPECTS and symptom-to-door time. Only standard-workflow-patients that arrived in our hospital with an NIHSS ≥7 while the angiography-suite was not occupied were included in the case-matched analysis in order to simulate a similar scenario for matching purposes. The maximum allowed difference for case-matching was chosen arbitrarily and was 10 years for age, six points for NIHSS, 3 points for ASPECTS and 45 min for symptom-to-door time. Continuous variables were compared between one-stop management and optimized workflow patients either by *t*-test, in the case of normal distribution, or by the Wilcoxon test, in the case of non-normal or ordinal distribution. Categorical variables were compared between the 2 groups by the Fisher’s exact test. The probability of favorable outcome (modified Rankin scale (mRS) ≤ 2) between the two groups at 90 days was further assessed by logistic regression using selected variables. Statistical analyses were performed with the MedCalc Statistical Software version 18 (MedCalc Software bv, Ostend, Belgium; http://www.medcalc.org; 2018).

## 3. Results

Two-hundred-thirty one-stop managed patients were included in our study (123 women; median age of 78 years (IQR 69–84)). The overall admission NIHSS was 15 (IQR 12–19) and 166/230 (72%) patients were diagnosed with an LVO, 25/230 (11%) with an SVO, 24/230 (10%) with an ICH, 11/230 (5%) with a Todd’s paresis and 4/230 (2%) with a recanalized LVO after transfer, respectively (Table 1). One-hundred-twenty-seven out of 230 (55%) cases were direct admissions, while 103/230 (45%) were transfer patients from a peripheral stroke center with a confirmed LVO. Of the 127 direct admission patients 74/127 (58%) were LVOs, 19/127 (15%) were SVOs, 23/127 (18%) were ICHs and 11/127 (9%) were Todd’s paresis. Of the 103 transfer patients, 61/103 (59%) received IV rtPA at the peripheral stroke center (“drip and ship”), 1/103 (1%) was diagnosed with a new subdural hematoma on FDCT that was not present on the initial external MDCT and 4/103 (4%) showed complete revascularization on baseline FDCTA at our center. The median time required between the external MDCT and the FDCT was 124 min (110–155; 90th percentile 218).

The overall door-to-FDCT time was 15 min (IQR 10–20; 90th percentile 26) and door-to-IVrtPA was 22 min (IQR 20–30; 90th percentile 41). The median door-to-groin time for LVO patients was 29 min (IQR 22–39; 90th percentile 50) with a median door-to-reperfusion time of 72 min (IQR 58–91; 90th percentile 117; Table 2). Patients with an M1 occlusion had a median door-to-reperfusion time of 64 min (IQR 56–87; 90th percentile 102). Any hemorrhage was depicted in 25/166 (15%), a parenchymal hematoma type-2 in 2/166 (1%) and a symptomatic intracranial hemorrhage (sICH) in 6/166 (4%) of the LVOs on follow-up imaging. Overall mortality was 22%. A favorable outcome was documented in 65/166 (39%) of the LVO patients at discharge. Nineteen LVO patients were lost to follow-up; favorable outcome and mortality for mothership patients with a pre-stroke mRS less than three were 57% and 31% while overall favorable outcome and mortality of mothership LVO patients were 51% and 32% respectively at 90 days after stroke onset.

**Figure 1:**
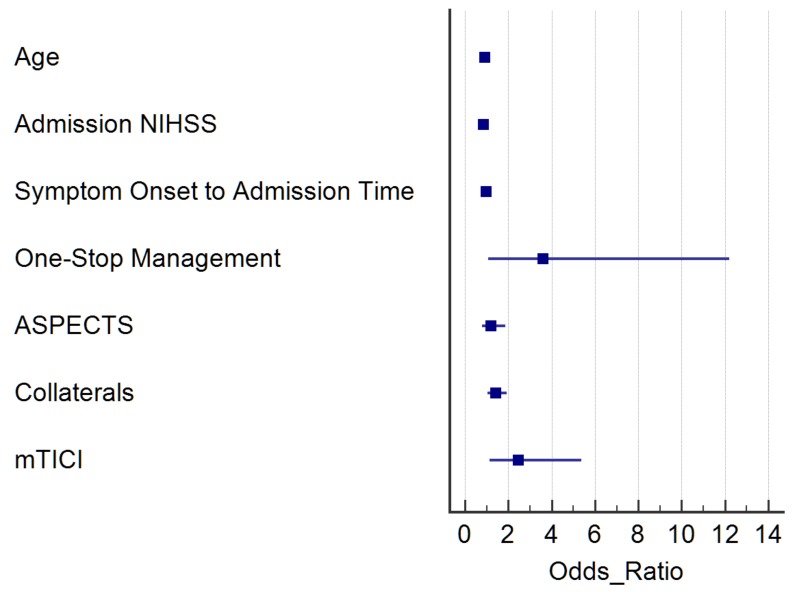
Logistic regression model comparing predictors of favorable clinical outcome in the matched population (one-stop management vs. normal management with MDCT).

The case-control analysis revealed 43 LVO matches for each group (one-stop vs. traditional management). Matching variables were not significantly different between one-stop and traditional workflow patients; other baseline and imaging characteristics (e.g., collaterals) were also balanced between the two groups (Table 3). We observed a significant reduction of door-to-groin and door-to-reperfusion times, both during working and off-duty hours, for direct admission and transfer patients. Safety variables, such as sICH, any hemorrhage on follow-up imaging or mortality were comparable between the two groups. Median discharge and 90 d mRS in the one-stop group was three (IQR 1–5) and two (IQR 1–5), respectively. The rate of good functional outcome at 90 days was significantly higher in the one-stop management group with 58% (25/43) as compared to 33% (14/43) in the normal workflow group (*p* = 0.029). In the logistic regression model comparing predictors of favorable clinical outcome in the matched population, the one-stop management (odds ratio (OR) 3.75; 95% confidence interval (CI) 1.13–12.44; *p* = 0.031) and successful reperfusion (OR 2.58; 95% CI 1.19–5.55; *p* = 0.015) were significant contributors to the prediction of a favorable outcome (Figure 1).

## 4. Discussion

Our observational study establishes on a large scale the results from prior brief reports with median door-to-groin times under 30 min and median door-to-reperfusion times under 90 min for patients triaged with a one-stop management [8]. The proposed fast and commercially available protocol is safe for both direct admission and transfer patients. Intravenous rtPA was administered in 63% of our patients, with rates of any hemorrhage, symptomatic ICH and mortality comparable to larger trials. Regarding outcome results it should be noted that our observational study had no restrictions regarding pre-stroke mRS or initial ASPECTS. Compared to the thrombectomy trial from 2015 with focus on rapid endovascular treatment we observed markedly lower door-to-groin times with a 90th percentile of 50 min in our study vs. 147 min in the ESCAPE trial [6]. Door-to-reperfusion times were also markedly lower in our study with a 90th percentile of 117 min compared to 190 min in the ESCAPE trial [6]. Even in comparison to recent trials published in 2018 and performed in large-volume centers with daily training and standardized workflows, our median intrahospital times were more than 30 min lower with 29 min door-to-groin time (106 min in the 3D-Separator trial) and 59 min imaging-to-reperfusion time (97 min in the DEFUSE 3 trial) in our study [14,15].

In the period from 2014 to 2015 we were able to significantly reduce intrahospital times from a median door-to-groin time of 121 to 64 min by standardizing interdisciplinary operating procedures, conducting frequent team meetings, and providing mutual feedback [11]. However, despite the increased workload and training in the consecutive years we were not able to further reduce the intrahospital times through the MDCT-route [16]. The current case-matched analysis shows, indeed, that a one-stop management allows for and additional reduction of intrahospital times (Table 3). The decreased handling times together with the increased rates of successful reperfusion in the one-stop arm patient pool led to a significant increase in rates of favorable functional outcome at 90 days.

While the increased reperfusion rates are probably a result of increased use of sophisticated thrombectomy techniques [17], the earlier groin puncture times may also play a role in increased rates of complete reperfusion in one-stop patients. A recent study of the HERMES dataset has shown a decreased probability of successful reperfusion with prolonged intrahospital times in LVO patients [18]. Another interesting aspect of the one-stop management in stroke is the interaction of IV rtPA and remaining thrombi after incomplete thrombectomy. In the meta-analysis of the five positive thrombectomy trials in 2015, the median door-to-needle time was 35 min and the median door-to-reperfusion time 148 min [2]. As the thrombolytic effect of IV lysis is rapidly lost after termination of the 60 min infusion there is usually no effect of IV rtPA on distal emboli after incomplete mechanical reperfusion. In our setting, both the IV rtPA infusion and thrombectomy are initiated within a narrow time frame which frequently leads to substantial reperfusion prior to completion of the IV rtPA infusion. This fact could lead to resolution of emboli in new territories or distal emboli with a positive effect on functional outcome.

**Figure 2:**
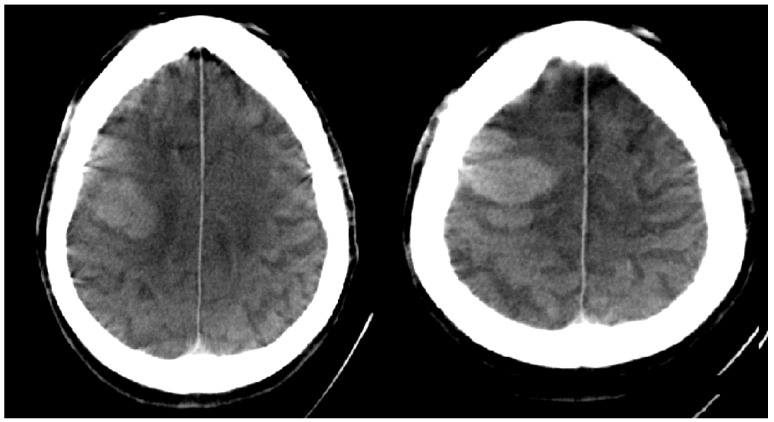
FDCT scan of intracranial hemorrhage which was missed by resident during off-hours.

In order to establish a one-stop management of stroke patients with the proposed protocol there is obviously a need for angio-time capacity within the stroke center. Angiography suite capacity should not be a problem in off-hours. We did not encounter a relevant problem in our effort to establish a one-stop management as more than half of the interventions performed in our center are mechanical thrombectomies. For other centers with limited angiography availability, possible solutions include the installation of a dedicated stroke angiography or a pre-notification system. Even with a dedicated stroke angiography-suite there is always the possibility of another stroke patient arriving while performing mechanical thrombectomy on the ‘stroke machine’. Regarding workload and safety, it should be noted that we decided to involve the senior physician in the exclusion of an ICH on FDCT images at all times, after a case of profound ICH which was missed by the resident on duty during off-hours (Figure 2). Discrepancies between residents and seniors in the interpretation of overnight head CTs and the detection of hemorrhages have been studied before for MDCT. The reported frequency of 1/230 cases in our study is comparable to the 141/22,590 cases in a study by Vagal et al. [19]. The workload of the stroke angiography can also be influenced by the NIHSS threshold chosen for the one-stop management. As of January 2017, we lowered our one-stop threshold to an admission NIHSS of ≥7 as a recent publication suggested that the best predictor for LVO is the NIHSS score with the aforementioned cut-off [20]. Other possibilities for a one-stop management include the combination of MDCT and C-arm in one room (so called MIYABI system) or the use of a mobile C-arm within the CT room [21]. Both systems have the disadvantages of a monoplanar angio system. The first solution has an additional drawback due to the increased costs for two machines, while with the second option the usually heavily utilized CT scanner is being blocked during thrombectomy. Our door-to-groin times were slightly higher (29 min) compared to the time metrics reported by Ribo et al. (17 min) and Jovin et al. (22 min). However, their door-to-reperfusion times (73 and 66 min respectively) were similar to ours (72 min) [22,23]. Furthermore, as we used non-invasive FDCT angiography for the delineation of LVOs, no unnecessary groin punctures were performed compared to a reported rate of 7% by Ribo et al. [24].

The main limitation of our study is the observational single-center design. All time metrics are prospectively documented in a stroke database, but the documentation is not performed in a blinded fashion. As this study was performed in a comprehensive stroke center, the number of LVO may be increased compared to regional stroke centers, which leads to an increased number of ischemic strokes compared to other centers. However, based on the observations including a significant reduction of door-to-groin times and secure triage of patients with hemorrhagic strokes, we have started a prospective, randomized trial in order to prove the effectiveness and safety of the proposed one-stop protocol.

## 5. Conclusions

One-stop management of stroke patients with a modern, FDCT-supporting angiography suite is feasible and allows for significantly shorter intrahospital times.

## Figures and Tables

**Table 1:** Clinical, angiographic, and procedural details of the 100 one-stop-management patients.

	All, *n* = 230	LVO, *n* = 166	SVO, *n* = 25	ICH, *n* = 24	Todd’s, *n* = 11	RLVO, *n* = 4
Age, median (IQR)	78 (69–84)	77 (68–84)	79 (71–87)	78 (75–83)	79 (73–82)	81 (77–86)
Admission NIHSS	15 (12–19)	16 (13–19)	13 (11–15)	15 (10–17)	12 (11–15)	13 (11–13)
Female	123 (54%)	90 (54%)	12 (48%)	12 (50%)	5 (46%)	4 (100%)
IV–rtPA	144 (63%)	112 (68%)	23 (92%)	1	4 (36%)	4 (100%)
Hemorrhage on initial FDCT	25 (11%)	1 (1%)	0 (0%)	24 (100%)	0 (0%)	0 (0%)
Occlusion site						
ICA-T		41 (25%)				
M1		88 (53%)				
M2		15 (9%)				
Other		22 (13%)				
Tandem occlusion		34 (21%)				
**Times, min** **(IQR; 90th Percentile)**						
Symptom to door	130 (70–195; 253)	154 (67–205; 264)	82 (66–134; 205)	105 (69–129; 204)	131(95–146; 186)	229 (181–259)
Door to FDCT	15 (10–20; 26)	14 (9–19; 25)	16 (12–24; 32)	17 (14–22; 30)	21 (13–23; 31)	16 (14–16)
Door to IV–rtPA	22 (20–30; 41)	22 (20–29; 38)	26 (20–45; 53)			
Door to treatment start^α^				21 (18–33; 34)	27 (19–32; 37)	
Door to groin		29 (22–39; 50)				
Groin to reperfusion		40 (28–60; 80)				
FDCT to reperfusion		59 (45–82; 101)				
Door to reperfusion		72 (58–91; 117)				
Door to reperfusion M1		64 (56–87; 102)				
extCT to FDCT		124 (110–155; 218)				
Direct admission	127 (55%)	74 (45%)	19 (76%)	23 (96%)	11 (100%)	0 (0%)
Working hours^β^	95 (41%)	68 (41%)	13 (52%)	11 (46%)	2 (18%)	1 (25%)
Reperfusion, mTICI2b-3		142 (86%)				
Any hemorrhage on FU	49 (21%)	25 (15%)	0 (0%)	24 (100%)	0 (0%)	
PH-2 hematoma on FU		2 (1%)				
sICH		6 (4%)				
Discharge NIHSS	5 (2–10)	5 (2–12)	7 (4–10)	5 (1–9)	4 (1–6)	6 (4–7)
Discharge mRS	4 (1–5)	4 (1–5)	4 (2–5)	2 (2–6)	3 (1–4)	3 (3–4)
Mortality	45/230 (20%)	36 (22%)	2 (8%)	5 (30%)	2 (18%)	0 (0%)
90 d mRS		4 (1–6)				
90 d favorable outcome		54/147 (37%)				

LVO, large vessel occlusion; SVO, small vessel occlusion; ICH, intracranial hemorrhage; RLVO, recanalized LVO during transfer; IQR, interquartile range, NIHSS, National Institutes of Health Stroke Scale; IV-rtPA, intravenous recombinant tissue plasminogen activator; FDCT, flat-detector CT; extCT, external CT in primary stroke center; mTICI, modified thrombolysis in cerebral infarction score; FU, follow-up; mRS, modified Rankin scale; α, intravenous injection of antihypertensive drugs in case of ICB or sedative drugs in case of seizures; β, weekdays 08:00 to 17:00.

**Table 2:** Procedural details of one-stop-management patients with large vessel occlusion.

Direct Admission *n* = 74	Min (IQR; 90th Percentile)
Door to FDCT	15 (12–20; 24)
Door to IV–rtPA	22 (20–29; 38)
Door to groin	34 (28–45; 51)
Groin to reperfusion	41 (26–55; 73)
FDCT to reperfusion	61 (47–81; 93)
Door to reperfusion	76 (61–92; 116)
Door to reperfusion of M1	68 (58–89; 101)
Occluded vessel	ICA-T 13 (18%), M1 42 (58%), M2 9 (12%)
Tandem occlusions	15 (20%)
**Transfer patients *n* = 92**	
extCT to FDCT	124 (110–155; 218)
Door to FDCT	10 (8–17; 25)
Door to groin	25 (19–33; 41)
Groin to reperfusion	38 (29–65; 87)
FDCT to reperfusion	56 (44–86; 110)
Door to reperfusion	68 (53–90; 126)
Door to reperfusion of M1	59 (52–84; 118)
Occluded vessel	ICA-T 28 (30%), M1 46 (50%), M2 6 (7%)
Tandem occlusions	19 (20%)
**Working hours *n* = 68**	
Door to FDCT	12 (7–16; 21)
Door to IV–rtPA	22 (20–26; 34)
Door to groin	25 (19–33; 41)
Groin to reperfusion	38 (25–53; 85)
FDCT to reperfusion	61 (42–69; 101)
Door to reperfusion	66 (52–85; 105)
**Off-hours *n* = 98**	
Door to FDCT	15 (10–21; 27)
Door to IV–rtPA	23 (19–29; 38)
Door to groin	33 (25–42; 60)
Groin to reperfusion	38 (25–53; 86)
FDCT to reperfusion	52 (42–69; 101)
Door to reperfusion	66 (52–85; 105)

**Table 3:** Case-control study of FDCT vs. MDCT patients, *n* =86.

	MDCT, *n* = 43	FDCT, *n* = 43	*p*-Value
Age median (IQR) *	*77 (69–81)*	*77 (69–82)*	*0.962*
Admission NIHSS *	*17 (14–20)*	*16 (13–20)*	*0.796*
CT ASPECTS *	*8 (7–9)*	*9 (7–10)*	*0.138*
Onset to door, min (IQR; 90th) *	*129 (76–200; 244)*	*160 (74–202; 221)*	*0.511*
Female	26 (61%)	26 (61%)	1.000
IV-rtPA	36 (84%)	30 (70%)	0.201
Hypertension	35 (81%)	33 (77%)	0.791
Hyperlipidemia	14 (33%)	20 (47%)	0.266
PAD	2 (5%)	5 (12%)	0.433
DM	11 (26%)	17 (40%)	0.249
Collateral grading	7 (5–8)	7 (4–8)	0.699
Direct admissions	30 (70%)	18 (42%)	**0.016**
Working hours	22 (51%)	19 (44%)	0.666
Door to CT, min (IQR; 90th)	15 (11–20; 24)	9 (6–14; 16)	**<0.001**
Door to IV-rtPA	27 (22–34; 35)	19 (12–22; 34)	**0.016**
Door to groin	60 (48–68; 79)	25 (19–30; 38)	**<0.001**
Working hours	60 (42–65; 85)	21 (17–25; 41)	**<0.001**
Off-hours	62 (53–69; 75)	25 (21–32; 38)	**<0.001**
Direct admissions	61 (54–67; 83)	26 (25–38; 44)	**<0.001**
Transfer patients	40 (30–69; 75)	21 (19–26; 35)	**<0.001**
Groin to reperfusion	42 (27–62; 94)	43 (33–60; 78)	0.866
CT to reperfusion	84 (71–99; 144)	59 (44–75; 96)	**<0.001**
Door to reperfusion	102 (85–117; 166)	68 (53–89; 104)	**<0.001**
Working hours	102 (79–145; 191)	62 (52–81; 104)	**0.006**
Off-hours	103 (93–116; 126)	74 (55–90; 109)	**<0.001**
Direct admissions	103 (85–121; 184)	72 (58–87; 103)	**0.001**
Transfer patients	102 (68–109; 120)	64 (51–88; 108)	**0.05**
ICA-T	7 (16%)	13 (30%)	0.179
M1	26 (61%)	25 (58%)	0.888
M2	9 (21%)	3 (7%)	0.117
Tandem occlusion	6 (14%)	7 (16%)	1
Successful reperfusion (mTICI2b-3)	31 (72%)	38 (88%)	0.102
sICH	3 (7%)	2 (5%)	1
Any hemorrhage	11 (26%)	7 (16%)	0.427
PH–2 hemorrhage	1 (2%)	1 (2%)	1
Discharge mRS	4 (2–5)	3 (1–5)	0.374
90d mRS	4 (1–5)	2 (1–5)	0.153
90d mRS of 0–2	14 (33%)	25 (58%)	**0.029**
Mortality	9 (21%)	10 (23%)	1

* Matching variables.
